# Smart Data: Where the Big Data Meets the Semantics

**DOI:** 10.1155/2017/6925138

**Published:** 2017-02-26

**Authors:** Trong H. Duong, Hong Q. Nguyen, Geun S. Jo

**Affiliations:** ^1^Institute of Science and Technology of Industry 4.0, Nguyen Tat Thanh University, Ho Chi Minh City, Vietnam; ^2^International University and Vietnam National University, Ho Chi Minh City, Vietnam; ^3^Inha University, Incheon, Republic of Korea

Big data technology is designed to address the challenges of the three Vs of big data, including* volume* (massive amount of data),* variety* (a range of data types and sources), and* velocity* (speed of data in and out). Big data is often captured without a specific purpose, leading to most of it being task-irrelevant data. The most important feature of data is neither the volume nor the other Vs, but its* value*. While big data is the technological foundation for data-driven business decision-making, smart data is an organized way to semantically compile, manipulate, correlate, and analyze different data sources.

To deal with the* volume*, the semantics technology facilitates better decision-making by converting massive amount of data into abstraction, meanings, and insights. Neural network algorithms offer advantages for deep learning and exploit the whole, rather than parts, of the data. The article* “A New Data Representation Based on Training Data Characteristics to Extract Drug Name Entity in Medical Text”* by M. Sadikin et al. proposes three data representation techniques to analyze the characteristics of word distribution and word similarities as a result of word-embedding training. These techniques include multilayer perceptrons, deep-network classifiers (deep belief networks, stacked denoising encoders), and long short term memory. In the article* “Objects Classification by Learning-Based Visual Saliency Model and Convolutional Neural Network”* by N. Li et al., a neuroscience-inspired classification method is proposed to simulate the human visual information processing mechanism. This method combines both visual attention model and convolutional neural network to increase the accuracy of classifying objects, especially in biology. S. Bi et al. propose a force-directed method using a fracture mechanic model to learn word embedding in the article* “Fracture Mechanics Method for Word Embedding Generation of Neural Probabilistic Linguistic Model.”* The method aims to improve the accuracy, recall, and text visualization of traditional language models, and a word embedding, a semantic vector representation, could be generated via the neural linguistic model.

For the* variety*, integrating heterogeneous data sources requires effective methods for providing well-defined ontologies and natural language processing. In the article* “A Character Level Based and Word Level Based Approach for Chinese-Vietnamese Machine Translation” *by P. Tran et al., a hybrid method is proposed to translate one natural language to another (e.g., from Chinese to Vietnamese) by combining strengths of statistics-based and rule-based translation approaches at both character and word levels. In addition to using bilingual corpora, this method takes advantage of translation and word-reordering capabilities of the statistical machine translation and the translation accuracy of the rules. The approach in the article* “N-Gram-Based Text Compression” *by V. H. Nguyen et al. presents an efficient method for compressing texts (in Vietnamese) by using *n*-gram dictionaries. This approach improves compression ratio and compression and decompression times compared with other methods.

To address the* velocity*, the ontology evolution techniques support dynamically, flexibly, and adaptively creating models of new objects, concepts, and relationships and using them to better understand new cues in the data that capture rapidly evolving events and situations. The article* “Social Media Meets Big Urban Data: A Case Study of Urban Waterlogging Analysis”* by N. Zhang et al. proposes a transfer-learning method to analyze urban waterlogging disasters in traffic operations management. It uses social media and satellite data; analyzes the correlation between severity, road networks, terrain, and precipitation; and adopts a multiview discriminant transfer-learning method to transfer knowledge among cities, as effectively applied in some cities in China and India. The article* “Automatic Construction and Global Optimization of a Multisentiment Lexicon”* by X. Yang et al. proposes an automatic construction and a global optimization framework of a multisentiment lexicon based on constraints of coordinate offsets. The method performs statistical training on a large corpus using neural network model, implements a sentiment disambiguation algorithm (based on word distribution density to distinguish the sentiment polarities in different contexts), and further integrates various human-annotated resources to learn the 10-dimensional sentiment lexicon for the optimization.

Different approaches are proposed to solve different problems in various areas. They aim to be accurate, actionable, and agile to feed smarter decision-making. Smart data harnesses the 3V-challenges and adopts semantics and neuroscience on the data to extract its* value*, the meeting point of the big data and the semantics.

*Trong H. Duong* *Hong Q. Nguyen* *Geun S. Jo*

